# Prognostic impact of palpable prostate tumors on disease progression after robot-assisted radical prostatectomy: a single-center experience

**DOI:** 10.1007/s11701-023-01669-w

**Published:** 2023-07-24

**Authors:** Antonio Benito Porcaro, Sebastian Gallina, Alberto Bianchi, Alessandro Tafuri, Emanuele Serafin, Andrea Panunzio, Giovanni Mazzucato, Rossella Orlando, Francesco Ditonno, Paola Irene Ornaghi, Riccardo Rizzetto, Clara Cerrato, Vincenzo De Marco, Matteo Brunelli, Salvatore Siracusano, Maria Angela Cerruto, Alessandro Antonelli

**Affiliations:** 1 Department of Urology, University of Verona, Azienda Ospedaliera Universitaria Integrata, Piazzale Stefani 1, 37126 Verona, Italy; 2 Department of Urology, Vito Fazzi Hospital, Lecce, Italy; 3 Department of Pathology, University of Verona, Azienda Ospedaliera Universitaria Integrata, Verona, Italy; 4 Department of Life, Health and Environmental Science, University of L’Aquila, L’Aquila, Italy

**Keywords:** Prostate cancer, Robot-assisted radical prostatectomy, Palpable prostate tumors, Digital rectal exam, Prostate cancer progression

## Abstract

**Objective:**

This study aimed to evaluate the impact of palpable prostate tumors on digital rectal exam (DRE) on the disease progression of prostate cancer (PCa) treated with RARP surgery in a tertiary referral center.

**Materials and methods:**

Overall, 901 patients were evaluated in a period ranging from January 2013 to October 2020. In the surgical specimen, unfavorable pathology included ISUP grade group ≥3, seminal vesicle invasion (SVI), and pelvic lymph node invasion (PLNI). Disease progression was defined as the occurrence of biochemical recurrence and/or local recurrence and/or distant metastases; its association with the primary endpoint was evaluated by Cox’s proportional model.

**Results:**

Palpable prostate tumors were detected in 359 (39.8%) patients. The overall median (IQR) follow-up was 40 months (17–59). PCa progressed in 159 cases (17.6%). Nodularity or induration of the prostate at DRE was significantly associated with features of unfavorable pathology, increased risk of PCa progression (hazard ratio, HR = 1.902; 95% CI: 1.389–2.605; *p* < 0.0001) and, on multivariable analysis, was an independent prognostic factor for disease progression after adjusting for clinical and pathological variables.

**Conclusions:**

Prostate tumors presenting with an abnormal DRE finding have an independent adverse outcome for disease progression after PCa surgery. They provide also independent prognostic information, as they may be more aggressive than impalpable PCa.

**Supplementary Information:**

The online version contains supplementary material available at 10.1007/s11701-023-01669-w.

## Introduction

In the aging male, prostate-specific antigen (PSA) testing and digital rectal exam (DRE) are pivotal elements in the diagnosis of prostate cancer (PCa) [1, 2]. The disease may present as a palpable abnormality in asymptomatic men [1, 2]. Also, DRE is pivotal for PCa primary clinical staging, as recommended by guidelines and TNM system [1, 2]. Palpable tumors may present at different local substages, which have implications on assessing clinical risk classes based on the D’Amico’s classification system [1, 2]. Clinical T stage only refers to DRE findings and local imaging findings are not considered in the actual TNM classification system [1, 2]. Although Radical Prostatectomy (RP) and External Beam Radiotherapy (EBRT) are recommended options for active treatment of PCa, between 27% and 53% of all patients develop rising PSA that precedes metastatic or disease progression, and will impact either disease-specific or overall mortality [1, 2]. According to a large systemic review and metanalysis, PCa progression is predicted by either clinical or pathological factors; however, the role of clinical local tumor stage, based on the DRE findings, is yet not clearly assessed [3]. This study aimed to evaluate the impact of palpable prostate tumors presenting as abnormal DRE findings on disease progression of PCa treated with Robot-Assisted Radical Prostatectomy (RARP) in a tertiary referral center.

## Materials and methods

### Data collection, patient selection, and tumor evaluation

The Institutional Review Board approval of the Azienda Ospedaliera Universitaria Integrata of Verona was obtained, and each patient provided informed consent for data collection and analysis. Data were collected prospectively but evaluated retrospectively. In a period ranging from January 2013 to October 2020, 1143 patients with clinically localized PCa were treated with RARP. Analysis was performed on 901 patients having available follow-up. Prostate-specific antigen (PSA; ng/mL), age (years), body mass index (BMI; kg/m^2^), prostate volume (PV; mL) and percentage of biopsy positive cores (BPC), the percentage ratio of positive and total taken cores (%), were evaluated for each case. PV was calculated by TRUS standard method. Biopsies performed elsewhere were assessed for the number of cores taken, tumor grade, and PV, which was measured by the trans-rectal approach. Clinical staging was assessed by the 2017 version of the TNM system (8th edition) with clinical T stage only referring to digital rectal exam findings. Patients were classified into risk classes, according to EAU guidelines [1]. Preoperative physical status was evaluated by the American Society of Anesthesiologists (ASA) system [4]. RARP was performed by experienced surgeons. Extended pelvic lymph nodes dissection (ePLND) was performed according to guidelines [1, 2]. Dissected lymph nodes were submitted in separate containers according to a standard anatomical template including external iliac, internal iliac + obturator, Marcille’s common iliac, and Cloquet’s nodal stations, bilaterally [5]. Specimens including prostate and dissected lymph nodes were placed in formalin and evaluated by dedicated pathologist. Prostates were weighted and tumors were graded according to the International Society of Urologic Pathology (ISUP) system [1, 2]. Tumor quantitation was assessed as tumor load (TL), which was defined as the percentage of prostate involved by cancer; specifically, our dedicated pathologist assessed tumor quantitation by visual estimation of all the glass slides after all microscopically identifiable foci of carcinoma have been circled with a marked pen, according to ISUP association [6]. Surgical margins were considered positive when cancer invaded the inked surface of the specimen. Removed lymph nodes were counted and assessed for cancer invasion. Prostate surgical specimens were staged by the 2017 version of the TNM system (8th edition) [1, 2]. Perioperative outcomes were evaluated for operating time, estimated intraoperative blood losses, nerve-sparing surgery, high- and low-volume surgeons, length of hospital stay (LOHS), and hospital readmission after discharge. Postoperative complications, which were monitored for a period of at least 3 months, were coded according to the Clavien–Dindo system [1, 2, 5].

### Evaluating the prognostic impact of palpable prostate tumors

Abnormal DRE findings at diagnosis were considered palpable tumors. DRE findings were related to the risk of PCa progression, which was defined as an unfavorable event including biochemical recurrence and/or local recurrence and/or distant metastases. The potential prognostic impact of palpable prostate tumors was also evaluated for unfavorable pathological sub-categories including ISUP ≥3, extracapsular extension (ECE), SVI, and PLNI. Decisions of further treatments after surgery or at disease progression were taken in a multidisciplinary setting including urologists, radiation oncologists, and oncologists trying to optimize recommendations with patient’s issues [1, 2]. After surgery, androgen deprivation therapy (ADT) was delivered in 154 cases (17.1%) and radiotherapy (RT) in 162 (17.9%) patients, of whom 77 (8.5%) with salvage purpose.

### Statistical methods

Continuous variables were measured for medians and interquartile ranges (IQR). Categorical factors were assessed for frequencies and rates (percentages). Associations of clinical and pathological factors with palpable prostate tumors were assessed by the Mann–Whitney test for continuous variables as well as by the Chi-squared test for categorical factors (univariate analysis). The length of time between surgery and the clinical outcome of interest (PCa progression) or the last follow-up was measured as time to event occurrence. Univariate and multivariate Cox proportional hazards models estimated the association of palpable prostate tumors presenting as abnormal DRE findings and other factors with the risk of PCa progression; hazard ratios (HR) and relative 95% confidence intervals (CI) were evaluated. Models were also evaluated for unfavorable pathological subgroups. Appropriate survival risk curves were generated. The software used to run the analysis was IBM-SPSS version 26. All tests were two sided with *p* < 0.05 considered to indicate statistical significance.

## Results

### Demographics and oncological outcomes

According to EAU criteria, 237 patients were considered low risk (26.3%), 479 (53.2%) intermediate risk, and 185 (20.5%) high risk. Palpable prostate tumors were detected in 359 (39.8%) patients. The overall median (IQR) follow-up was 40 months (17–59). PCa progressed in 159 cases (17.6%). Deaths occurred in 12 patients (1.3%), of whom 4 (0.4%) related to PCa progression. Population and subgroup demographics are reported in Table 1. Clinical factors associated with increased risk of PCa progression in palpable tumors were PSA, BPC ≥50%, ISUP Grade Group, cN1 and abnormal DRE (HR = 1.902; 95% CI: 1.389–2.605; *p* < 0.0001), as illustrated in Supplementary material: Figure 1. In the surgical specimen, progressing PCa patients were more likely to have positive surgical margins and unfavorable pathology including high-grade tumors, ECE, SVI, or PLNI.

**Table 1:** Risk of prostate cancer (PCa) progression in 901 patients treated with robot-assisted radical prostatectomy (RARP)

	Population	No PCa progression	PCa progression	Univariate analysis	
				HR (95% CI)	*p*-Value
Number (%)	901	742 (82.4)	159 (17.6)		
Clinical factors					
Age	65 (60–70)	65 (60–70)	65 (61–70)	1.024 (1.000–1.049)	0.053
BMI (kg/m^2^)	25.7 (23.9–28.1)	25.8 (23.9–28.1)	25.6 (24–28)	0.991 (0.942–1.043)	0.73
ASA 1	77 (8.5)	59 (8)	28 (11.3)	1	
ASA 2	743 (82.5)	615 (82.9)	128 (80.5)	0.671 (0.418–1.077)	0.098
ASA 3	81 (9.0)	68 (9.2)	13 (8.2)	0.762 (0.379–1.532)	0.445
PV	40 (30–50)	40 (30–50)	39 (30–50)	1.003 (0.994–1.012)	0.550
PSA < 10 ng/mL	731 (81.1)	631 (85)	100 (62.9)	1	
PSA : 10–20 ng/mL	132 (14.7)	92 (13.4)	40 (25.2)	2.524 (1.746–3.650)	<0.0001
PSA > 20 ng/mL	38 (4.2)	19 (2.6)	19 (11.9)	4.291 (2.620–7.030)	<0.0001
BPC < 50%	668 (74.1)	572 (77.1)	96 (60.4)	1	
BPC ≥50%	233 (25.9)	170 (22.9)	63 (39.6)	2.225 (1.616–3.062)	<0.0001
cT1	542 (60.2)	456 (61.5)	86 (54.1)	1	
cT2/3	359 (39.8)	286 (38.5)	73 (45.9)	1.902 (1.389–2.605)	<0.0001
ISUP 1	343 (38.1)	300 (40.4)	43 (27)	1	
ISUP 2/3	446 (49.5)	366 (49.3)	80 (50.3)	2.653 (3.893)	<0.0001
ISUP 4/5	112 (12.4)	76 (16.2)	36 (22.6)	4.447 (2.843–6.954)	<0.0001
cN0	851 (95.4)	708 (95.4)	143 (89.9)	1	
cN1	50 (5.5)	34 (4.6)	16 (10.1)	3.300 (1.959–5.559)	<0.0001
Pathological factors					
PW	50 (40–64)	50 (41.7–64.2)	53 (42–64)	1.004 (0.096–1.012)	0.346
TL	19 (10–30)	15 (10–30)	20 (15–40)	1.023 (1.015–1.031)	<0.0001
ISUP = 1	111 (12.3)	106 (14.3)	5 (3.1)	1	
ISUP = 2/3	600 (66.6)	518 (69.8)	82 (51.6)	5.201 (2.106–12.843)	<0.0001
ISUP = 4/5	190 (21)	118 (15.9)	72 (45.3)	15.743 (6.348–39.039)	<0.0001
pT2	706 (78.4)	618 (83.3)	88 (55.3)	1	
ECE	88 (9.8)	66 (8.9)	22 (13.8)	1.934 (1.211–3.088)	0.006
SVI	107 (11.9)	58 (7.8)	49 (30.8)	4.193 (2.950–5.959)	<0.0001
R0	685 (76)	586 (79)	99 (62.3)	1	
R1	216 (24)	156 (21)	60 (37.7)	1.279 (1.651–3.146)	<0.0001
pN 0/x	830 (92.1)	710 (95.7)	120 (75.5)	1	
pN1	71 (7.9)	32 (4.3)	39 (24.5)	5.549 (3.828–8.045)	<0.0001

Continuous variables are reported as medians (*IQR* interquartile ranges) and categorical factors as frequencies (percentages); *HR* hazard ratio; *CI* confidence interval; see “Materials and methods” section for abbreviations

### Adverse prognostic impact of palpable prostate tumors on disease progression

In the surgical specimen, palpable tumors were associated with features of high tumor load and unfavorable pathology including high primary tumor stage and PLNI, as reported in Table 2. On multivariate analysis, abnormal DRE was an independent prognostic factor for disease progression after adjusting for clinical and pathological variables, as detailed in Table 3. Interestingly, abnormal DRE remained an independent predictor also in subgroups with adverse pathology such as ISUP ≥3, extra-prostatic extension (ECE, SVI), and PLNI with hazard ratios higher than those for the entire cohort. Supplementary material: Figures 2 and 3 illustrate the prognostic impact of palpable prostate tumors at DRE on disease progression in unfavorable pathological subgroups including SVI and PLNI. The median risk of disease progression was 45 months for SVI and PLNI, which was shorter than the overall population (85 months), as explained in Supplementary material: Figure 1.

**Table 2:** Associations of clinical and pathological factors with palpable prostate tumors in 901 patients treated with robot-assisted radical prostatectomy

	Normal DRE	Abnormal DRE	Univariate analysis
			*p*-Value
Number (%)	542 (60.2)	359 (39.8)	
Clinical factors			
Age (years)	65 (60–70)	65 (61–70)	0.220
BMI (kg/m^2^)	26 (24.1–28.1)	25.4 (23.7–28.1)	0.200
ASA score 1	53 (9.8)	24 (6.7)	0.166
ASA score 2	445 (82.1)	298 (83)	
ASA score 3	44 (8.1)	37 (10.3)	
PSA < 10 (ng/mL)	449 (82.8)	282 (78.6)	0.158
PSA 10–20 (ng/mL)	75 (13.8)	57 (15.9)	
PSA < 20 (ng/mL)	18 (3.3)	20 (5.6)	
PV (mL)	40 (31–51)	38.7 (30–49.5)	0.076
BPC < 50%	425 (78.4)	243 (67.7)	<0.0001
BPC ≥ 50%	117 (21.6)	116 (32.3)	
ISUP = 1	243 (44.8)	100 (27.9)	<0.0001
ISUP = 2/3	264 (48.7)	182 (50.7)	
ISUP = 4/5	35 (6.5)	77 (21.4)	
cN0	527 (97.2)	324 (90.3)	<0.0001
cN1	15 (2.8)	35 (9.7)	
Pathological factors			
PW (gr)	52 (42–65)	50 (41–62)	0.066
TL (%)	15 (10–25)	20 (10–30)	<0.0001
ISUP = 1	89 (16.4)	22 (6.1)	<0.0001
ISUP = 2/3	372 (68.4)	228 (63.5)	
ISUP = 4/5	81 (14.9)	109 (30.4)	
IPD (pT2)	455 (83.9)	251 (69.9)	<0.0001
ECE (pT3a)	40 (7.4)	48 (13.4)	
SVI (pT3b)	47 (8.7)	60 (16.7)	
R0	422 (77.9)	263 (73.3)	0.113
R1	120 (22.1)	96 (26.7)	
pNx/0	516 (95.2)	314 (87.5)	<0.0001
pN1	26 (4.8)	45 (12.3)	

Continuous variables are reported as medians (*IQR* interquartile ranges) and categorical factors as frequencies (percentages); *DRE* digital rectal exam; normal DRE, non-palpable prostate tumors; abnormal DRE, palpable prostate tumors; see “Materials and methods” section for abbreviations

**Table 3:** The prognostic role of palpable prostate tumors at digital rectal exam (DRE) in predicting disease progression after robot-assisted radical prostatectomy

Endpoint	Unadjusted HR (95% CI)	Clinical adjusted HR (95% CI)	Pathological adjusted HR (95% CI)
Population (*n* = 901)			
Non-palpable tumors	1.000	1.000	1.000
Palpable tumors	1.902 (1.389–2.605)	1.512 (1.092–2.094) (*)	1.497 (1.084–2.066) (**)
Pathology ISUP ≥3 (*n* = 439)			
Non-palpable tumors	1.000	1.000	1.000
Palpable tumors	1.751 (1.217–2.519)	1.476 (1.016–2.144) (*)	1.612 (1.113–2.333) (***)
Extracapsular extension (*n* = 88)			
Non-palpable tumors	1.000	1.000	1.000
Palpable tumors	3.262 (1.251–8.505)	5.515 (1.534–19.833) (*)	5.354 (1.840–15.583) (^)
Seminal vesicle invasion (*n* = 107)			
Non-palpable tumors	1.000	1.000	1.000
Palpable tumors	2.278 (1.270–4.083)	2.639 (1.322–5.231) (*)	1.870 (1.021–3.425) (^^)
Pelvic lymph node invasion (*n* = 71)			
Non-palpable tumors	1.000	1.000	1.000
Palpable tumors	2.609 (1.255–5.421)	2.906 (1.302–6.487) (*)	2.763 (1.176–6.482) (^^^)

*HR* odds ratio; *CI* confidence interval

(*): after adjusting for age, BMI, ASA, PV, PSA, BPC, ISUP, cN; (**): after adjusting for ISUP, pT, R1, pN1; (***), after adjusting for pT, R, pN; (^); after adjusting for ISUP, R, pN; (^^), after adjusting for ISUP, R, pN; (^^^), after adjusting for ISUP, pT, R; according to DRE findings, normal DRE is non-palpable tumors, while abnormal DRE are palpable cancers; see also Tables 1 and 2 as well as “Materials and methods” section for details

## Discussion

Although primary treatments for localized PCa are performed with curative intent, the risk of biochemical recurrence (BCR) is not negligible, involving from 27% to 53% of patients [1, 2].

Moreover, about 30% of PSA recurring patients after RP will develop clinical recurrence and among them, 16.4% will die of disease progression [1, 2]. According to a large metanalysis, PCa metastatic progression was predicted by positive surgical margins, pathology tumor grade, PSA doubling time, and early salvage RT for patients treated with RP as well as by biopsy tumor grade, tumor clinical stage, and interval to BCR for cases treated with RT [3]. Therefore, EAU proposed a new BCR model after RP, which stratified patients into low- and high-risk classes with the former having a PSA doubling time longer than 1 year and/or pathology Gleason score <8; the model was validated externally and reached independent predictor status not only for metastatic progression but also for PCa-specific mortality; however, the discriminative ability of the model was moderate [7]. So far, the prognostic potential of the primary tumor stage defined by DRE findings in patients treated with RP remains understudied. Halpern and associates have shown that suspicious DRE was an independent risk factor for detecting clinically significant PCa across all PSA levels in a large trial including 35,350 men who underwent DRE in the screening arm of the prostate, lung, colorectal, and ovarian cancer screening trial, which considered Gleason score ≥7 as clinically significant PCa; moreover, investigators concluded that DRE demonstrated prognostic usefulness for PSA >3 ng/mL [8]. Our study has demonstrated that abnormal DRE at clinical presentation is a negative prognostic factor of disease progression after RP; moreover, it reached independent predictive status for both the overall population and unfavorable pathological subgroups after adjusting for other variables. Although the results of our study are important for clinical practice, confirmatory studies are awaited.

In PCa patients presenting with abnormal DRE and surgically treated, a critical drawback is the detection of unfavorable pathology, which includes ISUP grade ≥3, ECE, SVI, and PLNI [1, 2]. Unfavorable pathology is the main predictor of disease progression after surgery, and it becomes a critical drawback in the low- and intermediate-clinical risk categories, which are different for EAU and NCCN systems [1–3, 7, 9, 10]. The role of DRE is still underestimated; other factors are required for stratifying patients in each risk class and to decide which treatment to adopt, especially in those patients initially managed with active surveillance (AS) [1, 2, 9, 10].

Moreover, anatomopathological staging may be inaccurate due to lymphatic drainage variability that can extend beyond standard surgical margins [11].

Our study has shown that abnormal DRE findings were positively associated with features of unfavorable disease including high tumor load, extra-prostatic extension, and PLNI. So far, adverse pathology was confirmed to be a negative prognostic factor of PCa progression and its association with abnormal DRE is a subject awaiting confirmatory studies**.**

Our findings stress the importance of this simple and practical tool for counseling and clinical managing of PCa.

When DRE results are abnormal, PCa presents as an irregular, firm, or hard nodule, which may have a variable extension within the gland. When the tumor seems organ confined, patients are classified with the clinical T2 stage, which is stratified into further categories according to the extension through the gland [1, 2]. When palpable tumors show periprostatic extension, they are categorized as stage T3 stage; finally, PCa may present as a large palpable abnormality associated with features of local extensive disease, which defines the cT4 category [1, 2].

A nodularity or induration at DRE seems to be an independent prognostic factor of PCa progression and this finding could generate hypotheses on PCa biology. Prostate tumors probably become palpable when they are located at the peripheral zone of the gland and reach a sufficient volume to be detected as an abnormal finding at DRE. Although 70% of these cancers arise from the peripheral zone of the gland, only 40% or less become palpable at clinical presentation. We speculate that palpable prostate tumors may result from a high-density growing pattern of the cancer cell population. The overall process is probably enhanced by local stimulating factors produced by the tumor itself.

Our study has several limitations. First, it was retrospective and single center. Second, the follow-up period was short. Third, the overall and cancer-specific survival were not evaluated because of the limited number of patients involved in these events. Last, confirmatory studies are missing. Our study has also strengths. The primary outcome was evaluated at disease progression, which is a stronger endpoint than only BCR. Procedures were performed by both low- and high-volume surgeons who did not bias staging results, thus reflecting real-world practice in tertiary referral centers.

## Conclusions

Prostate tumors presenting as abnormal DRE findings have an independent adverse outcome for disease progression after PCa surgery. Palpable prostate tumors, while providing independent prognostic information, might have more aggressive growth patterns than impalpable cancers.

## Supplementary Information

Below is the link to the electronic supplementary material.

Figure 1 Risk curves of time to prostate cancer (PCa) progression stratified by palpability of tumors at digital rectal examination (DRE) in 901 patients including all risk classes according to European Association of Urology (EAU) and treated with robot assisted radical prostatectomy (RARP). On univariate analysis (Cox’s proportional hazards), the risk of disease progression was unfavorable for abnormal DRE palpable tumors (hazard ratio, HR=1.902; 95% CI:1.389–2.605; *p* < 0.0001). Supplementary file 1 (JPG 45 KB)

Figure 2 Risk curves of time to PCa progression comparing palpable and impalpable prostate tumors at DRE in patients with adverse pathology in the surgical specimen including seminal vesicle invasion in 107 cases. Median time to PCa progression of palpable tumors was 45 months, which was significantly lower (67 months) compared to controls with normal DRE (HR = 2.278; 95% CI:1,270–4.083; *p* = 0.006), according to Cox’s univariate proportional hazards. Supplementary file 2 (JPG 46 KB)

Figure 3 Risk curves of time to PCa progression comparing palpable and non-palpable prostate tumors at DRE in patients with adverse pathology in the surgical specimen including pelvic lymph node invasion in 71 cases. Median time to PCa progression of palpable tumors was 45 months, which was significantly lower (69 months) compared to controls with normal DRE (HR = 2.609; 95% CI:1,255–5.421; *p* = 0.010), according to Cox’s univariate proportional hazards. Supplementary file 3 (JPG 45 KB)

## Data Availability

The data presented in this study are available on request from the corresponding author. The data are not publicly available due to ethical reasons.
