# Supplementary figures and images for: Prognostic impact of palpable prostate tumors on disease progression after robot-assisted radical prostatectomy: a single-center experience
Source: J Robot Surg. 2023 Jul 24;17(5):2471–7. doi: 10.1007/s11701-023-01669-w (PMC10492703; doi:10.1007/s11701-023-01669-w)

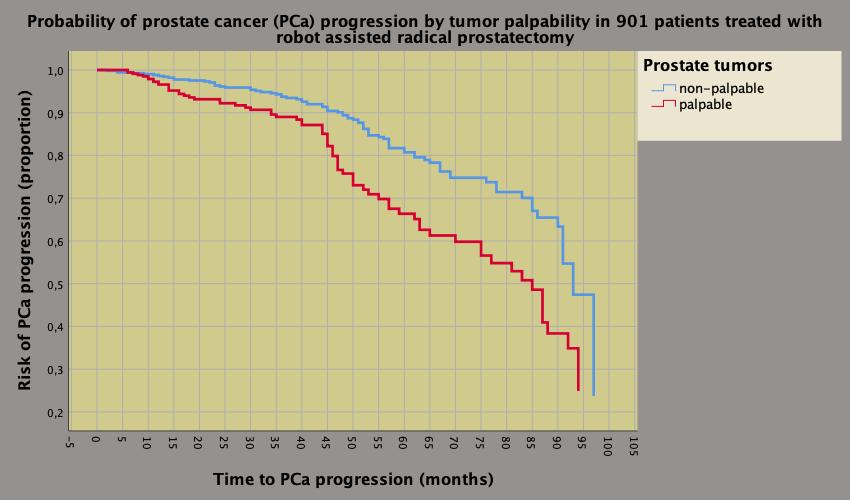

Supplement: Supplementary file 1 — Risk curves of time to prostate cancer (PCa) progression stratified by palpability of tumors at digital rectal examination (DRE) in 901 patients including all risk classes according to European Association of Urology (EAU) and treated with robot assisted radical prostatectomy (RARP). On univariate analysis (Cox’s proportional hazards), the risk of disease progression was unfavorable for abnormal DRE palpable tumors (hazard ratio, HR=1.902; 95% CI:1.389–2.605; p < 0.0001). Supplementary file 1 (JPG 45 KB) [file 11701_2023_1669_MOESM1_ESM.jpg]

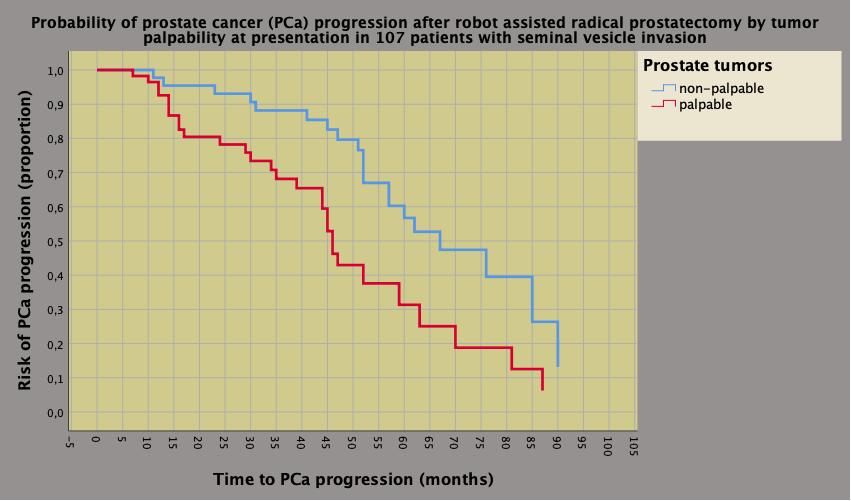

Supplement: Supplementary file 2 — Risk curves of time to PCa progression comparing palpable and impalpable prostate tumors at DRE in patients with adverse pathology in the surgical specimen including seminal vesicle invasion in 107 cases. Median time to PCa progression of palpable tumors was 45 months, which was significantly lower (67 months) compared to controls with normal DRE (HR = 2.278; 95% CI:1,270–4.083; p = 0.006), according to Cox’s univariate proportional hazards. Supplementary file 2 (JPG 46 KB) [file 11701_2023_1669_MOESM2_ESM.jpg]

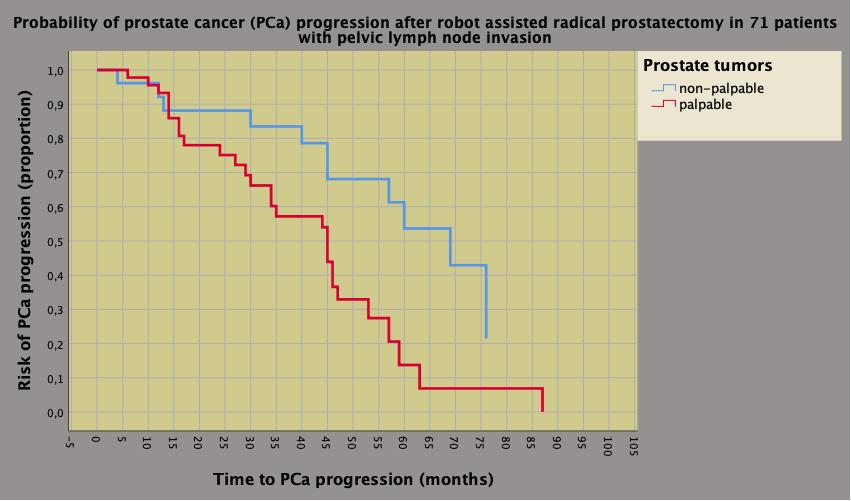

Supplement: Supplementary file 3 — Risk curves of time to PCa progression comparing palpable and non-palpable prostate tumors at DRE in patients with adverse pathology in the surgical specimen including pelvic lymph node invasion in 71 cases. Median time to PCa progression of palpable tumors was 45 months, which was significantly lower (69 months) compared to controls with normal DRE (HR = 2.609; 95% CI:1,255–5.421; p = 0.010), according to Cox’s univariate proportional hazards. Supplementary file 3 (JPG 45 KB) [file 11701_2023_1669_MOESM3_ESM.jpg]
